# Medical Literature

**Published:** 1848

**Authors:** G. N. Fitch

**Affiliations:** Professor of the Principles and Practice of Medicine in Rush Medical College


ARTICLE III.

*Medical Literature.* By G. N. Fitch, M.D., Professor of the Principles and Practice of Medicine in Rush Medical College.

Amid the much good and valuable daily accruing from the almost universal disposition on the part of our profession to record the results of their observation and experience, not a little of decided evil tendency is found. All the numerous volumes, essays, and monographs annually issued from the medical press have their readers; all exercise some influence for good or evil over at least a portion of those readers. There are said to be more "false facts than false theories" in medicine, and the publication of either adds nothing to our practical knowledge — to that knowledge which we may render useful at the bed-side — but greatly retards its acquisition. They put us, like a false finger board at a cross road, upon the wrong track, and it may be only after long and vainly groping our way in error that it is discovered. Young practitioners are particularly liable to be thus misled. The consequence to them may be but a loss of time and professional reputation; a loss from which future care and watchfulness may enable them to recover. But how with their patients? The detection of the error has been at their expense — an expense of health, mayhap life! One writer relates false facts, and proceeds to base a particular hypothesis upon them which would be legitimately deducible, *if the asserted facts were really such;* but which, in the absence of true principles to sustain it, cannot lead to other than erroneous, probably mischievous practice. Another relates facts in themselves true, but which form exceptions to the ordinary pathology or symptoms of the particular disease. He proceeds to build upon them his hypothetical superstructure, and lay down his rules of treatment — treatment which may be found applicable to, and beneficial in, the tenth or exceptional case, but inert or fatally active in the nine, or numerous cases forming the rule. Another, first perfects his theory and then casts about for props upon which to lean it — for facts to uphold it. He may, like the one above, find these in exceptional cases, or he may, by ingenious sophistry, torture others to his support. Another advocates, by *post hoc propter hoc* reasoning, a routine practice into which himself has fallen in a particular disease. The disease may be one, the tendency of which is to spontaneous termination in health under any treatment short of that which would be in itself destructive; this termination, however, is liable to be hastened by appropriate treatment, or delayed from its want. If the practice recommended is inert, it will be fortunate for such subjects of the disease as fall to the charge of those adopting it. To such invalids the question is one, not of life or death, or even of present safety, with impaired health, but of time and present suffering only. But this "routine practice" may, and too often does, consist of treatment in all cases of a disease and in all its stages, under which, and mayhap in spite of which, a few have recovered, but which is "destructive" of life or health to most. If such practice is ingeniously urged, and cases cited to sustain it, while its mischievous effects are not observed or not related, it can scarcely fail to be adopted to a considerable extent.

Yet all these writers claim and expect that to which, as writers and authors, as those to whom young physicians look for correct information and correct views, they are presupposed to be entitled, viz: credence for their statements, applause for their theories, and confidence in the remedial means to which those theories point. And rarely are they entirely disappointed. As before said, they all have their readers, and some portion of those readers is influenced by them. Still farther to strengthen this influence, reviewers, who should be sentinels at the doors of the temple of medical science, to prevent the ingress of aught which could mislead its votaries, of aught wrong in fact or in inference, only more extensively disseminate every medical vagary, either with sweeping commendation, or with a charity here altogether misplaced, treat them as they would the character of a deceased, dwell strongly upon the good, and avoid any mention of the evil. The remedy is easy. I grant the reviewer should notice any and every medical theory, but he should notice them in all their relations of cause and effect. By so doing he retails erroneous ones, it is true, to many who might not otherwise see them, but he retails them as the apothecary does poison, with a label attached that all can read and be warned by, not as the empiric does his nostrum, with words of praise, leaving dear bought experience to detect their fatal tendency.

